# Report of the Commissioner of Education for the Year 1881

**Published:** 1883-12

**Authors:** 


**Report of the Commissioner of Education for the Year 1881.** Washington: Government Printing Office. 1883.

In view of the vast amount of material contained in these ponderous reports, it is not surprising that they appear so late. The present one contains nearly 1,000 pages. We find in the following, relative to medical schools, that which is instructive, in view of the present agitation for the better control of medical education:

The schools of medicine, dentistry and pharmacy, number 126, and are attended by 14,536 students. The instructors are 1,746 in number. The schools of medicine are styled regular, eclectic, and homœopathic. The regular schools number 76, and have 1,243 instructors and 10,250 students, of whom 989 are graduate students. The grounds, buildings and apparatus of these schools are valued at $2,208,470, and the receipts for last year from tuition and other fees were $375,493. The productive funds are small, $366,193; and the libraries are meagre, containing only 40,757 volumes. Eight eclectic schools have 80 instructors and 882 students; grounds, buildings and apparatus valued at $230,500, and receipts from tuition and other fees, $39,760. The homœopathic schools are 12, with 173 instructors and 1,285 students, $244,000 in grounds, buildings and apparatus, and an income of $39,224.

The subjects treated by the Commissioner in connection with these schools are courses preparatory to the study of medicine, entrance examinations, the prevalent form and amount of medical instruction, clinical instruction, graded courses, and the gratifying progress in medical education. The preparatory courses of Cornell University, the University of Pennsylvania, and Johns Hopkins University are described. The subjects and extent of entrance examinations are stated. The average requirements for a medical degree are that the student shall study medicine under some competent physician three years, attending, meanwhile, two courses of lectures in distinct years, the latter course in the institution from which the degree is sought. Thirteen schools are said to have graded courses of instruction. The studies of the first year are usually anatomy, physiology, histology, chemistry, and, less frequently, materia medica. Those of the second year are pathology, theory and practice of medicine, therapeutics and obstetrics. Special departments of anatomy and chemistry and of clinical medicine and surgery occupy the student in a number of schools. The studies of the third year are theory and practice, therapeutics, and obstetrics continued, diseases of women and children, surgery, ophthalmology, otology, mental and nervous diseases, and occasionally dermatology, laryngology, and medical jurisprudence. Several quotations given show that substantial progress has been made recently in medical education.

The number of degrees of all classes conferred in course was 12,093; honorary, 535. They were distributed as follows: In letters, 4,035 in course, 185 honorary; in science, 1,167 in course, 14 honorary; in philosophy, 476 in course, 49 honorary; in art, 29 in course, 2 honorary; in theology, 312 degrees and diplomas in course, 171 honorary degrees; *in medicine, 4,896 in course, 22 honorary;* in law, 1,002 in course, 92 honorary. The Commissioner says: "As a means of maintaining the full significance of scholastic honors one of two conditions should be made a requisite for degrees: First, a special examination; second, extended research or other worthy achievement in the department of knowledge represented by the degree. Our leading institutions insist more and more upon these requirements, and the relative proportion of honorary degrees decreases from year to year."

